# Mortality in children aged <5 years with severe acute respiratory illness in a high HIV-prevalence urban and rural areas of South Africa, 2009–2013

**DOI:** 10.1371/journal.pone.0255941

**Published:** 2021-08-12

**Authors:** Oluwatosin A. Ayeni, Sibongile Walaza, Stefano Tempia, Michelle Groome, Kathleen Kahn, Shabir A. Madhi, Adam L. Cohen, Jocelyn Moyes, Marietjie Venter, Marthi Pretorius, Florette Treurnicht, Orienka Hellferscee, Anne von Gottberg, Nicole Wolter, Cheryl Cohen

**Affiliations:** 1 Faculty of Health Sciences, Division of Epidemiology and biostatistics, School of Public Health, University of the Witwatersrand, Johannesburg, South Africa; 2 National Institute for Communicable Diseases of the National Health Laboratory Service, Centre for Respiratory Diseases and Meningitis, Johannesburg, South Africa; 3 Influenza Division, Centers for Disease Control and Prevention, Atlanta, Georgia, United States of America; 4 Influenza Programme, Centers for Disease Control and Prevention-South Africa, Pretoria, South Africa; 5 Mass Genics, Duluth, Georgia, Unites States of America; 6 Faculty of Health Sciences, Medical Research Council, Respiratory and Meningeal Pathogens Research Unit, University of the Witwatersrand, Johannesburg, South Africa; 7 Department of Science and Technology/National Research Foundation: Vaccine Preventable Diseases, University of the Witwatersrand, Johannesburg, South Africa; 8 Faculty of Health Sciences, MRC/Wits Rural Public Health and Health Transitions Research Unit (Agincourt), School of Public Health, University of the Witwatersrand, Johannesburg, South Africa; 9 Centre for Global Health Research, Umeå University, Umeå, Sweden; 10 INDEPTH Network, Accra, Ghana; 11 Department of Medical Virology, University of Pretoria, Pretoria, South Africa; 12 School of Pathology, University of the Witwatersrand, Johannesburg, South Africa; 13 Division of Infectious Diseases, Hubert Department of Global Health, Rollins School of Public Health, School of Medicine, Emory University, Atlanta, GA, United States of America; Public Health England, UNITED KINGDOM

## Abstract

**Background:**

Severe acute respiratory illness (SARI) is an important cause of mortality in young children, especially in children living with HIV infection. Disparities in SARI death in children aged <5 years exist in urban and rural areas.

**Objective:**

To compare the factors associated with in-hospital death among children aged <5 years hospitalized with SARI in an urban vs. a rural setting in South Africa from 2009–2013.

**Methods:**

Data were collected from hospitalized children with SARI in one urban and two rural sentinel surveillance hospitals. Nasopharyngeal aspirates were tested for ten respiratory viruses and blood for pneumococcal DNA using polymerase chain reaction. We used multivariable logistic regression to identify patient and clinical characteristics associated with in-hospital death.

**Results:**

From 2009 through 2013, 5,297 children aged <5 years with SARI-associated hospital admission were enrolled; 3,811 (72%) in the urban and 1,486 (28%) in the rural hospitals. In-hospital case-fatality proportion (CFP) was higher in the rural hospitals (6.9%) than the urban hospital (1.3%, p<0.001), and among HIV-infected than the HIV-uninfected children (9.6% vs. 1.6%, p<0.001). In the urban hospital, HIV infection (odds ratio (OR):11.4, 95% confidence interval (CI):5.4–24.1) and presence of any other underlying illness (OR: 3.0, 95% CI: 1.0–9.2) were the only factors independently associated with death. In the rural hospitals, HIV infection (OR: 4.1, 95% CI: 2.3–7.1) and age <1 year (OR: 3.7, 95% CI: 1.9–7.2) were independently associated with death, whereas duration of hospitalization ≥5 days (OR: 0.5, 95% CI: 0.3–0.8) and any respiratory virus detection (OR: 0.4, 95% CI: 0.3–0.8) were negatively associated with death.

**Conclusion:**

We found that the case-fatality proportion was substantially higher among children admitted to rural hospitals and HIV infected children with SARI in South Africa. While efforts to prevent and treat HIV infections in children may reduce SARI deaths, further efforts to address health care inequality in rural populations are needed.

## Introduction

Pneumonia is a leading cause of death in children <5 years of age globally, accounting for over 900,000 deaths annually and largely occurring in low- and middle-income countries (LMICs) [1]. Sub-Saharan Africa (SSA) is disproportionately affected, accounting for more than half of pneumonia deaths globally [1].

In South Africa, pneumonia and influenza together comprised the second leading cause of death in children aged <5 years in 2013, and was responsible for about 10% of deaths in this age group [2]. Reducing mortality in children aged <5 years to 25 per 1,000 live births is a target of the Sustainable Development Goal (SDG) [3]; however, reducing the burden of severe acute respiratory illness (SARI) remains a major challenge in Africa [1]. Possible approaches include improved access to vaccines such as pneumococcal conjugate vaccine (PCV), *Haemophilus influenzae* type b vaccine, reduction in HIV-mother to child transmission (MTCT), integrated management of childhood illness (IMCI), indoor air pollution reduction, and improved hospital care [4]. Despite improvements in patient survival and advances in the development of new antimicrobials and vaccines, pneumonia is still associated with high morbidity and mortality burden in children especially those living with HIV [5]. Previous analysis of surveillance data from South Africa and other LMICs suggested that SARI mortality is higher in rural compared to urban settings [6–9], possibly due to differences in access to public health services and facilities [10,11], underlying HIV prevalence and health conditions. Studies have shown that rural residents are more likely to travel more than 15 minutes to reach their health facilities compared with urban residents (62% vs. 24%, respectively) [11].

Studies on mortality among children aged <5 years hospitalized with SARI have been conducted in South Africa, but none have examined the differences in risk factors for SARI-related in-hospital death in urban and rural areas with high HIV prevalence [6,12]. We aimed to compare the in-hospital case fatality proportion and factors associated with in-hospital mortality among children aged <5 years hospitalized with SARI in a rural vs. an urban area with high HIV prevalence in South Africa from 2009 to 2013.

## Materials and methods

### Health care system in South Africa

In South Africa, there are four levels of the health care referral system. Level one is comprised of three steps: primary health care clinic (first step), community health care centre (second step), and district hospitals (third step). The regional hospital is the second level. Regional hospitals receive referrals and provide specialist treatment to district hospitals. The provincial tertiary hospital is the third level while the national central hospitals serve as the fourth level of referral [13,14].

### Study setting

We used SARI programme data from February 2009 through December 2013. Since 2009, the active, hospital-based SARI programme has been ongoing in three of South Africa’s nine provinces. For the present study, we limited our analysis to children aged <5 years enrolled in three of the four participating hospitals: one urban hospital (Chris Hani-Baragwanath Academic Hospital [CHBAH]) in Gauteng Province, and two rural hospitals (Matikwana and Mapulaneng Hospitals) in Mpumalanga Province. The fourth hospital was excluded because of its location in a peri-urban area and to allow direct comparison between the rural and urban settings.

CHBAH is a level 2 regional hospital with approximately 3200 beds, including 408 paediatric beds and approximately 32 paediatric and neonatal intensive care unit (ICU) beds. Mapulaneng Hospital is also a regional hospital with 297 beds and 3 ICU beds; none are specifically dedicated to paediatric admissions. Matikwana Hospital is a level 1 district hospital with 178 beds and no paediatric ICU beds [15]. Children admitted to Matikwana Hospital that require ICU facilities are referred to the provincial tertiary hospitals once the Mapulaneng Hospital ICU beds are fully occupied.

### Case definition

A case of SARI was defined as a hospitalized child aged <5 years with illness onset within 7 days of hospitalization meeting age-specific inclusion criteria as follows: children aged two days to ≤ 3 months with a physician’s diagnosis of acute lower respiratory tract infection (ALRTI) or sepsis, children aged 3 months to < 5 years with a physician’s diagnosis of ALRTI (which included pneumonia, bronchitis, pleural effusion and bronchiolitis). For the main analysis we excluded children admitted to the urban hospital short-stay ward for less than 48 hours because the two rural hospitals did not have a comparable short stay wards. Patients admitted to the short-stay ward are usually less severe than those admitted to the medical wards, potentially introducing biases if included in the main analysis.

### Study procedures

The SARI programme methods have been published previously [16]. Briefly, hospitalized individuals residing in the hospital’s catchment area admitted between Mondays through Friday during the study period were screened for enrolment. Beginning in January 2013, enrolment was limited to only two days per week at CHBAH to conserve resources. The number of hospitalized children meeting the study case definition was documented throughout the surveillance period. From enrolled children, data on socio-demographic factors, clinical presentation, and underlying conditions were obtained through hospital record review and structured interviews were conducted by trained research staff within two days of admission. Beginning in 2012, child weight, height and mid upper arm circumference were collected to assess malnutrition. Based on the WHO weight-for-age z-score, we considered children with a z-score < -2 standard deviation as underweight [17].

### Laboratory methods

Whole blood and nasopharyngeal aspirates (NPA) specimens were collected from enrolled children and transported to the National Institute for Communicable Diseases (NICD) within 72 hours of collection. Multiplex real-time reverse-transcription PCR (RT-PCR) assays were used to test the NPAs for 10 respiratory viruses (influenza A and B viruses, adenovirus, parainfluenza virus 1, 2 and 3, enterovirus, respiratory syncytial virus (RSV), human metapneumovirus and rhinovirus) [18]. Blood was tested for *S*.*pneumoniae* using a single-target (*lytA*) quantitative real-time-PCR assay. If clinician initiated, HIV testing was performed according to the standard protocol [19]. Otherwise, dried blood spot specimens were tested by PCR for children aged <18 months and by ELISA for children aged ≥18 months. Clinician-requested CD4+ T-lymphocyte counts were determined by flow cytometry [20]. During 2009–2011, tuberculosis testing used sputum smear microscopy and culture. In 2011, GeneXpert was introduced for the diagnosis of pulmonary tuberculosis as part of the national tuberculosis programme [21].

### Statistical analysis

Race was defined as blacks vs. others, others being white, mixed race or Asian. Type of housing was categorized as brick, iron sheeting and others (mud, thatched); 2 or more doses of pneumococcal vaccine was labelled as “Yes” or “No” based on the number of doses of pneumococcal vaccine received. Yes “2 or more doses” while No “<2 doses”. We defined any other underlying illness as any of chronic lung disease, asthma, renal disease, heart disease, neurological disease and diabetes. The outcome of interest was in-hospital death which we interpreted as death during hospital admission vs. those that did not die during hospital admission.

#### Univariate analyses

Characteristics between children admitted at the urban and rural hospitals were compared using the Pearson’s Chi squared test. The comparison of children hospitalized at the urban and rural hospitals was repeated to include children admitted to the short stay-ward as a sensitivity analysis. Characteristics between HIV-infected and HIV-uninfected children were compared using chi squared test and univariate logistic regression analysis. We analysed the difference in percentage of HIV infected children by receipt of antiretroviral therapy (ART) by hospital site and year using Pearson Chi squared test. HIV-infected children were categorized into two immunosuppression categories: (i) mild immunosuppression (CD4+ T-lymphocytes ≥200/mm^3^ or equivalent age-appropriate CD4+ percentage for children aged <5 years), or (ii) severe immunosuppression (CD4+ T-lymphocytes <200/mm^3^ or equivalent age-appropriate CD4+ percentage for children aged <5 years) [22].

#### Multivariable analyses

We assessed factors associated with in-hospital death using multivariable logistic regression models, starting with all variables that were significant at p<0.1 on univariable analysis and dropping non-significant factors with stepwise backward selection. All pairwise interactions of factors significant at the final multivariable additive model were evaluated. Two-sided p-values <0.05 were considered significant. For each univariable analysis, we used all available case information. In the multivariable model, patients with missing data for included variables were dropped from the model.

To compare the characteristics of children who died at the urban hospital with those who died at the rural hospitals, we used a multivariable logistic regression model restricted to children that died. Similar to the in-hospital death analysis, factors significant in univariate analysis were included in the model. For all logistic regression analyses, tuberculosis infection, pneumococcus *lytA* PCR results on blood and malnutrition were evaluated only on univariate analysis due to the elevated number of missing data.

#### Survival analysis

Survival estimates were compared between those who died, looking at the interval from symptom onset to hospitalization, and date of hospitalization to date of death in the rural and urban hospitals using the Kaplan-Meier survival estimates with a log-rank test for equality of survival function. All analyses were performed using Stata version 14 (StataCorp Limited, College Station, Texas, United States of America).

#### Ethical consideration

The study was approved by the University of the Witwatersrand Human Research Ethics Committee (Medical) in December 2015 (Reference number: M151030). The protocol for SARI surveillance was approved by Ethics committee of University of the Witwatersrand. Annual recertification was obtained from the Ethics committee (Reference number: M140824). This surveillance was deemed non-research by the US Centers for Disease Control and Prevention (non-research determination number: 2012–6197). Written informed consent was obtained from parents, primary care givers or legal guardians of the children before enrolment.

## Results

**Fig 1:**
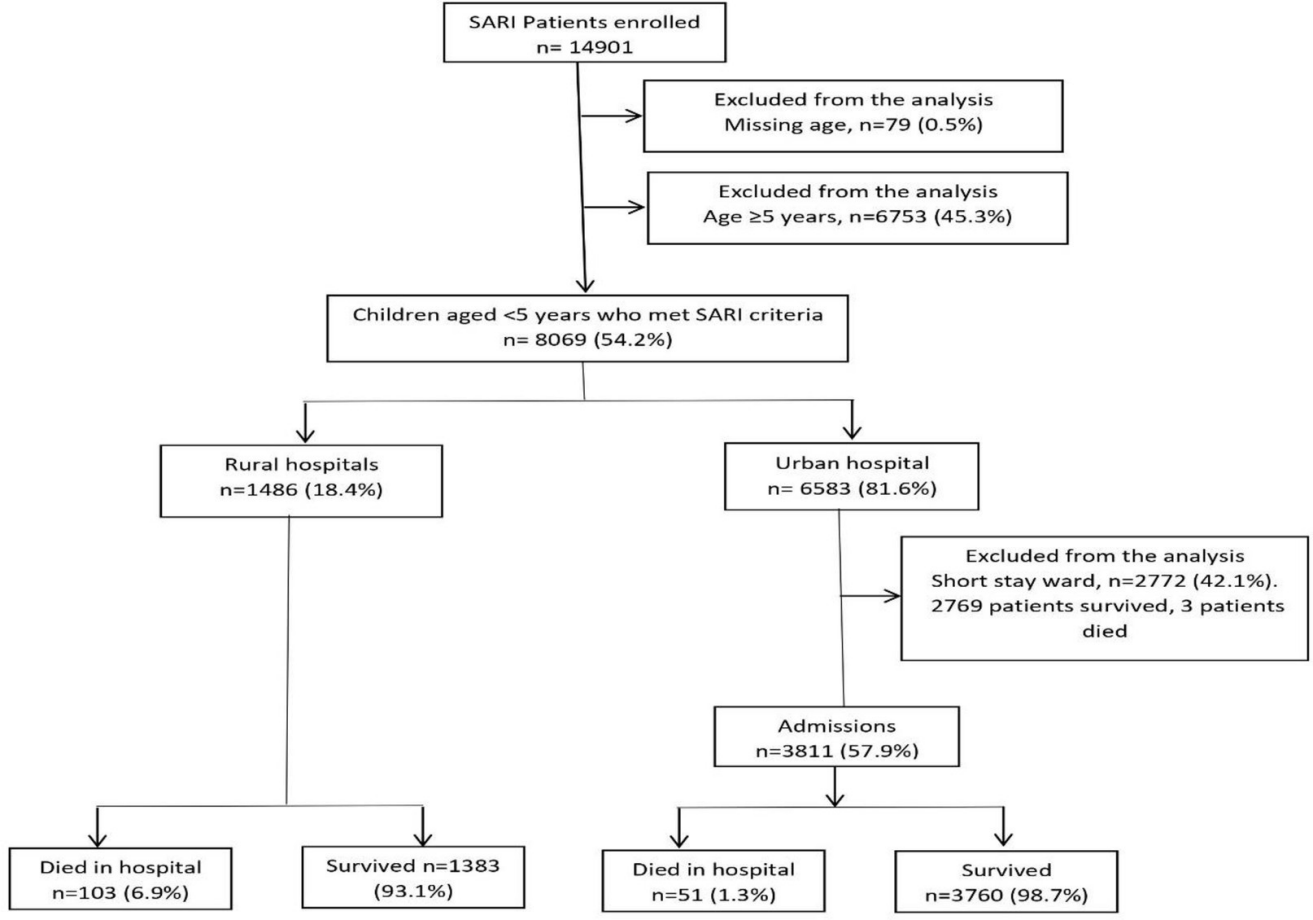
Flow chart of children enrolled in the South African severe acute respiratory illness (SARI) surveillance program at urban and rural hospitals in South Africa, 2009 to 2013.

From February 2009 through December 2013, 14,901 patients were enrolled into the SARI surveillance program in urban and rural hospitals. Of these, 8069 children who met SARI criteria were screened for enrolment; 6583/8069 (81.6%) were enrolled in the urban hospital and 1486/8069 (18.4%) in the rural hospitals. We excluded 2772 (42.1%) children admitted in the short stay ward of the urban hospital. A total of 5297 children were included in the main analysis, including 3811 (71.9%) from the urban hospital and 1486 (28.1%) from the rural hospitals (Fig 1). The characteristics of children admitted to the rural hospitals individually are shown in S1 Table.

### Characteristics of children hospitalized in the urban and rural hospitals

#### Demographic characteristics and clinical presentation

The urban hospital had a higher proportion of children hospitalized with SARI aged <1 year compared to the rural hospitals (74.9% vs. 58.8%, p<0.001) (Table 1). The majority of children were male (57.3%) and black race (98.2% in urban hospital vs. 99.5% in rural hospitals). The overall case-fatality proportion (CFP) was 2.9% and differed significantly by hospital; 103 (6.9%) children died in the rural hospitals compared with 51 (1.3%) in the urban hospitals (p<0.001). Differences in treatment were also observed with 53.7% receiving supplemental oxygen therapy in the urban hospital compared with 21.2% in the rural hospitals. Among children with pneumococcal vaccination data available, 1032/1386 (74.5%) at the urban hospital had received 2 or more doses of pneumococcal vaccine compared to 551/855 (64.4%) at the rural hospitals (p<0.001) (Table 1). A total of 1914 (36.1%) of the children were <14 weeks of age and were not eligible to receive 2 or more doses of pneumococcal vaccine. On sensitivity analysis, including all children admitted to the short stay ward at CHBAH, the case-fatality proportion was 0.8% (54/6583) at the urban hospital vs. 6.9% (103/1486) at the rural hospitals (p<0.001) (S2 Table).

**Table 1:** Comparison of the demographic characteristics, clinical presentation and respiratory pathogens detected among children aged <5 years hospitalized with SARI at urban and rural hospitals, South Africa 2009–2013.

Characteristics		Total	Urban hospital	Rural hospitals	P value
		N = 5297 (%)	N = 3811 (%)	N = 1486 (%)	
**Socio-demographics**					
Age group (years)	< 1	3729 (70.4)	2855 (74.9)	874 (58.8)	**<0.001**
	1–4	1568 (29.6)	956 (25.1)	612 (41.2)	
Sex	Female	2260 (42.7)	1619 (42.5)	641 (43.1)	0.666
	Male	3037 (57.3)	2192 (57.5)	845 (56.9)	
Race	Black	5203 (98.2)	3724 (97.7)	1479 (99.5)	**<0.001**
	Other	94 (1.8)	87 (2.3)	7 (0.5)	
2 or more doses of pneumococcal vaccine	Yes	1583 (70.6)	1032 (74.5)	551 (64.4)	**<0.001**
	No	658 (29.4)	354 (25.5)	304 (35.6)	
Type of housing	Bricks	4099 (77.4)	2680 (70.3)	1419 (95.5)	**<0.001**
	Iron sheeting	1086 (20.5)	1079 (28.3)	7 (0.5)	
	Others	112 (2.1)	52 (1.4)	60 (4.0)	
Number of people sleeping in a room	>2	4961 (94.8)	3511 (93.2)	1450 (99.0)	**<0.001**
	≤2	271 (5.2)	256 (6.8)	15 (1.0)	
**Clinical presentation and course**					
Symptoms prior to admission (days)	≥2	2885 (54.9)	2217 (58.6)	668 (45.3)	**<0.001**
	<2	2373 (45.1)	1567 (41.4)	806 (54.7)	
Antibiotics prescribed on admission	Yes	4915 (95.1)	3446 (93.5)	1469 (99.3)	**<0.001**
	No	251 (4.9)	241 (6.5)	10 (0.7)	
Supplementary oxygen therapy	Yes	2337 (44.5)	2025 (53.7)	312 (21.2)	**<0.001**
	No	2909 (55.5)	1747 (46.3)	1162 (78.8)	
Duration of hospitalization (days)	<5	2522 (48.2)	1800 (47.8)	722 (49.2)	0.336
	≥5	2712 (51.8)	1968 (52.2)	744 (50.8)	
In-hospital death	Yes	154 (2.9)	51 (1.3)	103 (6.9)	**<0.001**
	No	5143 (97.1)	3760 (98.7)	1383 (93.1)	
**Co-infections and underlying medical conditions**					
HIV status	Positive	490 (13.5)	257 (10.3)	233 (20.7)	**<0.001**
	Negative	3129 (86.5)	2236 (89.7)	893 (79.3)	
Tuberculosis infection	Yes	92 (9.8)	85 (10.4)	7 (5.7)	0.100
	No	846 (90.2)	730 (89.6)	116 (94.3)	
Malnutrition (reported)	Yes	33 (0.6)	26 (0.7)	7 (0.5)	0.382
	No	5255 (99.4)	3780 (99.3)	1475 (99.5)	
Malnutrition (Underweight)	Yes	308 (24.9)	209 (26.9)	99 (21.6)	**0.035**
	No	927 (75.1)	567 (73.1)	360 (78.4)	
*Any other underlying illness	Yes	256 (4.8)	195 (5.1)	61 (4.1)	0.126
	No	5033 (95.2)	3612 (94.9)	1421 (95.9)	
**Respiratory pathogens**					
Any respiratory virus	Yes	3960 (77.1)	2878 (77.6)	1082 (75.9)	0.189
	No	1175 (22.9)	831 (22.4)	344 (24.1)	
Pneumococcal infection on lytA PCR	Yes	166 (5.9)	113 (7.1)	53 (4.4)	**0.002**
	No	2637 (94.1)	1479 (92.9)	1158 (95.6)	

• Column percentage were calculated as a percent of all those with available data for the variables (i.e. not including missing).

• Variables statistically significant at p< 0.05 presented in boldface.

• *Any other underlying illness (any of chronic lung disease, asthma, renal disease, heart disease, neurological disease, diabetes).

• Missing values for covariates were as follows: 2 or more doses of pneumococcal vaccine (n = 3056), Number of people sleeping in a room (n = 65), Symptoms prior to admission (days) (n = 39), Antibiotics prescribed on admission (n = 131), Supplementary oxygen therapy (n = 51), Duration of hospitalization (days) (n = 63), HIV (n = 1678), Tuberculosis infection (n = 4359), Malnutrition (reported) (n = 9), Malnutrition (Underweight) (n = 4062), Any other underlying illness (n = 8), Any respiratory virus (n = 162), Pneumococcal infection on lytA PCR (n = 2494).

#### Underlying medical conditions

Information on HIV infection status was available for 2493 (65.4%) and 1126 (75.8%) of children from the urban and rural hospitals, respectively. Overall, 13.5% of children were HIV-infected and HIV infection was significantly higher among children admitted in the rural hospitals (20.7%) compared with the urban hospital (10.3%, p<0.001) (Table 1). A similar percentage of children were diagnosed with tuberculosis at the urban hospital compared to the rural hospitals (85/815 (10.4%) vs. 7/123 (5.7%), p = 0.100). A similar percent of children were reported as malnourished at the urban and rural site (26/3806 (0.7%) vs. 7/1482 (0.5%), p = 0.382). We were able to assess malnutrition for 1235/1795 (68.8%) of individuals enrolled during 2012–2013 using the z-score, among which 24.9% were underweight. A significantly higher proportion of children were underweight at the urban site as compared to the rural site (26.9% vs. 21.6%, p = 0.035) (Table 1).

#### Respiratory pathogens

A total of 5135 (96.9%) children were tested for respiratory viruses but, there was no significant difference between urban and rural hospitals for infection with any respiratory virus (77.6% in urban versus 75.9% in rural sites, p = 0.189). Overall, 2803 (52.9%) of children were tested for pneumococcal *lytA* in blood of which a significantly higher proportion tested positive at the urban site compared to the rural site (113/1592 (7.1%) vs. 53/1211 (4.4%), p = 0.002) (Table 1).

### HIV infection

Overall, 13.5% of children were HIV-infected (Table 1). Compared with HIV-uninfected children hospitalized for SARI, HIV-infected children were more likely to present for admission ≥2 days of symptom onset (odds ratio [OR]: 1.31; 95% confidence interval [95% CI]: 1.08–1.59, p = 0.006), to be admitted for ≥5 days (OR: 2.40, 95% CI: 1.95–2.95, p<0.001) and die in the hospital (OR: 6.67, 95% CI: 4.42–10.07, P<0.001) (Table 2). Infection with tuberculosis was not different by rural vs. urban (Table 1), and not different by HIV status in rural hospitals, but significantly different by HIV status in the urban hospital (S3 Table).

**Table 2:** Demographics and clinical characteristics of HIV-infected and HIV-uninfected children aged <5 years hospitalized with SARI at urban and rural hospitals, South Africa 2009–2013.

Characteristics		Total	HIV-infected	HIV-uninfected	Univariate analysis	P value
		N = 3619 (%)	N = 490 (%)	N = 3129 (%)	OR (95% CI)	
**Socio-demographics**						
Hospital	Urban	2493 (68.9)	257 (52.4)	2236 (71.5)	Reference	**<0.001**
	Rural	1126 (31.1)	233 (47.6)	893 (28.5)	2.27 (1.87–2.75)	
Age group (years)	<1	2536 (70.1)	313 (63.9)	2223 (71.0)	0.72 (0.59–0.88)	**0.001**
	1–4	1083 (29.9)	177 (36.1)	906 (29.0)	Reference	
Sex	Male	2087 (57.7)	248 (50.6)	1839 (58.8)	Reference	**0.001**
	Female	1532 (42.3)	242 (49.4)	1290 (41.2)	1.39 (1.14–1.68)	
Black	Others	57 (1.6)	8 (1.6)	29 (1.6)	Reference	0.912
	Black	3562 (98.4)	482 (98.4)	3080 (98.4)	0.96 (0.45–2.04)	
2 or more doses of pneumococcal vaccine	No	475 (29.2)	79 (35.6)	396 (28.1)	Reference	**0.023**
	Yes	1154 (70.8)	143 (64.4)	1011 (71.9)	0.71 (0.53–0.96)	
Number of people sleeping in a room	≤2	191 (5.3)	22 (4.5)	169 (5.5)	Reference	0.387
	>2	3381 (94.7)	464 (95.5)	2917 (94.5)	1.22 (0.78–1.93)	
**Clinical presentation and course**						
Symptoms prior to admission (days)	<2	1627 (45.2)	192 (39.5)	1435 (46.1)	Reference	**0.006**
	≥2	1969 (54.8)	294 (60.5)	1675 (53.9)	1.31 (1.08–1.59)	
Antibiotics prescribed on admission	No	158 (4.5)	11 (2.3)	147 (4.8)	Reference	**0.012**
	Yes	3379 (95.5)	471 (97.7)	2908 (95.2)	2.16 (1.16–4.02)	
Supplementary oxygen therapy	No	2024 (56.4)	255 (52.7)	1769 (57.0)	Reference	0.078
	Yes	1566 (43.6)	229 (47.3)	1337 (43.0)	1.19 (0.98–1.44)	
Duration of hospitalization (days)	<5	1690 (47.2)	142 (29.4)	1548 (49.9)	1.00 (Reference)	**<0.001**
	≥5	1893 (52.8)	341 (70.6)	1552 (50.1)	2.40 (1.95–2.95)	
In-hospital death	No	3523 (97.3)	443 (90.4)	3080 (98.4)	Reference	**<0.001**
	Yes	96 (2.7)	47 (9.6)	49 (1.6)	6.67 (4.42–10.07)	
**Co-infections and underlying medical conditions**						
Tuberculosis infection	No	624 (89.8)	95 (81.9)	529 (91.4)	Reference	**0.002**
	Yes	71 (10.2)	21 (18.1)	50 (8.6)	2.34 (1.34–4.07)	
Malnutrition (reported)	No	3595 (99.4)	482 (98.6)	3113 (99.6)	Reference	**0.005**
	Yes	20 (0.6)	7 (1.4)	13 (0.4)	3.48 (1.38–8.76)	
Malnutrition (Underweight)	No	639 (75.2)	45 (54.9)	594 (77.3)	Reference	**<0.001**
	Yes	211 (24.8)	37 (45.1)	174 (22.7)	2.81 (1.76–4.48)	
*Any other underlying illness	No	3441 (95.2)	462 (94.5)	2979 (95.3)	Reference	0.450
	Yes	175 (4.8)	27 (5.5)	148 (4.7)	1.18 (0.77–1.79)	
**Respiratory pathogens**						
Any respiratory virus	No	791 (22.1)	157 (32.4)	634 (20.5)	Reference	**<0.001**
	Yes	2792 (77.9)	328 (67.6)	2464 (79.5)	0.54 (0.44–0.66)	
Pneumococcal infection on lytA PCR	No	2257 (94.2)	322 (90.2)	1935 (94.9)	Reference	**<0.001**
	Yes	139/2396 (5.8)	35/357 (9.8)	104/2039 (5.1)	2.02 (1.35–3.02)	

• Column percentage were calculated as a percent of all those with available data for the variables (i.e. not including missing).

• Variables statistically significant at p< 0.05 presented in boldface. ORs unadjusted.

• *Any other underlying illness (any of chronic lung disease, asthma, renal disease, heart disease, neurological disease, diabetes).

• Missing values for covariates were as follows: 2 or more doses of pneumococcal vaccine (n = 1990), Number of people sleeping in a room (n = 47), Symptoms prior to admission (days) (n = 23), Antibiotics prescribed on admission (n = 82), Supplementary oxygen therapy (n = 29), Duration of hospitalization (days) (n = 36), Tuberculosis infection (n = 2924), Malnutrition (reported) (n = 4), Malnutrition (Underweight) (n = 2769), Any other underlying illness (n = 3), Any respiratory virus (n = 36), Pneumococcal infection on lytA PCR (n = 1223).

#### HIV-infected children and highly active antiretroviral therapy (HAART)

The HIV prevalence amongst hospitalized children decreased over the surveillance period from 19% in 2009 to 8% in 2013; however, the prevalence was consistently higher at the rural site compared to the urban site (Table 3). On sensitivity analysis, assuming that all the children with missing data on HIV status were HIV-uninfected, the prevalence of HIV amongst hospitalized children with SARI also decreased over the study period at both sites and was consistently higher at the rural site compared to the urban site (S4 Table). The percentage of HIV-infected children receiving antiretrovirals increased over the study period and was higher in the urban site initially. Only 129 (26.3%) HIV-infected children had available CD4+ T-lymphocyte data, of whom 83 (75.5%) in the urban and 9 (47.4%) in the rural hospitals had severe immunosuppression (CD4+ T-lymphocytes <200/mm3 or equivalent age-appropriate CD4+ percentage for children aged <5 years) (Table 3).

**Table 3:** Percentage of children that are HIV-infected, those receiving ARV and those having low CD4 count by year among children aged <5 years hospitalized with SARI at urban and rural hospitals, South Africa 2009–2013.

	HIV-infected children				HIV-infected children on ARV			Low CD4 count		
Year	Total	Urban site N = 3811	Rural site N = 1486	P value	Urban site N = 257	Rural site N = 233	P value	Urban site N = 110	Rural site N = 19	P value
		n/N (%)	n/N (%)		n/N (%)	n/N (%)		n/N (%)	n/N (%)	
2009	155/821 (19)	102/655 (16)	53/166 (32)	<0.001	34/78 (44)	5/26 (19)	0.001	31/40 (78)	1/1 (100)	0.162*
2010	135/765 (18)	62/503 (12)	73/262 (28)		16/37 (43)	9/28 (32)		20/26 (77)	2/4 (50)	
2011	72/733 (10)	37/513 (7)	35/220 (16)		7/23 (30)	7/13 (54)		9/15 (60)	0/2 (0)	
2012	90/818 (11)	44/559 (8)	46/259 (18)		13/26 (50)	13/23 (57)		16/20 (80)	4/7 (57)	
2013	38/482 (8)	12/263 (5)	26/219 (12)		5/8 (63)	10/18 (56)		7/9 (78)	2/5 (40)	
Overall	490/3619 (14)	257/2493 (10)	233/1126 (21)		75/172 (44)	44/108 (41)		83/110 (75)	9/19 (47)	
Missing data	1678	1318	360		85	125		62	89	

*Fisher’s exact test.

### Factors associated with in-hospital death

In the urban hospital, HIV infection (odds ratio (OR):11.4, 95% confidence interval (CI):5.4–24.1) and presence of any other underlying illness (OR: 3.0, 95% CI: 1.0–9.2) were the only factors independently associated with death. In the rural hospitals, HIV infection (OR: 4.1, 95% CI: 2.3–7.1) and age <1 year (OR: 3.7, 95% CI: 1.9–7.2) were independently associated with death, whereas duration of hospitalization ≥5 days (OR: 0.5, 95% CI: 0.3–0.8) and any respiratory virus detection (OR: 0.4, 95% CI: 0.3–0.8) were negatively associated with death (Table 4).

**Table 4:** Factors associated with in-hospital death among children aged <5 years hospitalized with SARI at urban and rural hospitals, South Africa 2009–2013.

	Urban hospital				Rural hospitals			
Characteristics	Hospitalized case fatality proportion (%)	Univariate analysis OR (95%CI)	Multivariable analysis OR (95%CI)	P value	Hospitalized case fatality proportion (%)	Univariate analysis OR (95%CI)	Multivariable analysis OR (95%CI)	P value
**Age group (years)**								
<1	42/2855 (1.5)	1.6 (0.8–3.2)			82/874 (9.4)	**2.9 (1.8–4.8)**	**3.7 (1.9–7.2)**	**<0.001**
1–4	9/956 (0.9)	Reference			21/612 (3.4)	Reference	Reference	
**Sex**								
Male	25/2192 (1.1)	Reference			43/845 (5.1)	Reference	NS	
Female	26/1619 (1.6)	1.4 (0.8–2.5)			60/641 (9.4)	**1.9 (1.3–2.9)**		
**Race**								
Other	6/87 (6.9)	Reference			1/7 (14.3)	Reference		
Black African	45/3724 (1.2)	**0.2 (0.1–0.4)**	NS		102/1479 (6.9)	0.4 (0.1–3.7)		
**2 or more doses of pneumococcal vaccine**								
No	7/354 (2.0)	Reference			26/304 (8.6)	Reference		
Yes	18/1032 (1.7)	0.9 (0.4–2.1)			31/551 (5.6)	0.6 (0.4–1.1)		
**Number of people sleeping in a room**								
≤ 2 people	4/256 (1.6)	Reference			1/15 (6.7)	Reference		
>2 people	46/3511 (1.3)	0.8 (0.3–2.3)			100/1450 (6.9)	1.0 (0.1–8.0)		
**Clinical presentation and course**								
**Duration of symptoms prior to admission**								
<2 days	17/1567 (1.1)	Reference			51/806 (6.3)	Reference		
≥2 days	33/2217 (1.5)	1.4 (0.8–2.5)			51/668 (7.6)	1.2 (0.8–1.8)		
**Antibiotics prescribed on admission**								
No	5/241 (2.1)	Reference			2/10 (20%)	Reference		
Yes	45/3446 (1.3)	0.6 (0.2–1.6)			101/1469 (6.9%)	0.3 (0.1–1.4)		
**Supplemental oxygen therapy**								
No	7/1747 (0.4)	Reference	Omitted		20/1162 (1.7)	Reference	Omitted	
Yes	43/2025 (2.1)	**5.4 (2.4–12.0)**			83/312 (26.6)	**20.7 (12.4–34.3)**		
**Duration of hospitalization (days)**								
<5	21/1800 (1.2)	Reference			61/722 (8.5)	Reference	Reference	**0.008**
≥5	30/1968 (1.5)	1.31 (0.7–2.3)			41/744 (5.5)	**0.6 (0.4–1.0)**	**0.5 (0.3–0.8)**	
**Co-infection and underlying medical conditions**								
**HIV status**								
Negative	13/2236 (0.6)	Reference	Reference	**<0.001**	36/893 (4.0)	Reference	Reference	**<0.001**
Positive	17/257 (6.6)	**12.1 (5.8–25.2)**	**11.4 (5.4–24.1)**		30/233 (12.9)	**3.5 (2.1–5.8)**	**4.1 (2.3–7.1)**	
**Tuberculosis infection**								
No	13/730 (1.8)	Reference			6/116 (5.2)	Reference	Omitted	
Yes	2/85 (2.4)	1.3 (0.3–6.0)			2/7 (28.6)	**7.3 (1.2–45.9)**		
**Malnutrition (reported)**								
No	49/3780 (1.3)	Reference	Omitted		99/1475 (6.7)	Reference	Omitted	
Yes	1/26 (3.9)	3.0 (0.4–22.9)			3/7 (42.9)	**10.4 (2.3–47.2)**		
**Malnutrition (Underweight)**								
No	2/567 (0.4)	Reference	Omitted		15/360 (4.2)	Reference	Omitted	
Yes	3/209 (1.4)	4.1 (0.7–24.8)			12/99 (12.1)	**3.2 (1.4–7.0)**		
*Any other underlying illness								
No	44/3612 (1.2)	Reference	Reference	**0.048**	94/1421 (6.6)	Reference	NS	
Yes	6/195 (3.1)	**2.6 (1.1–6.1)**	**3.04 (1.0–9.2)**		8/61 (13.1)	**2.1 (1.0–4.6)**		
**Respiratory pathogens**								
**Any respiratory virus**								
No	17/831 (2.1)	Reference	NS		35/344 (10.2)	Reference	Reference	**0.003**
Yes	33/2878 (1.2)	**0.6 (0.3–1.0)**			55/1082 (5.1)	**0.5 (0.3–0.7)**	**0.4 (0.3–0.8)**	
**Pneumococcal infection on lytA PCR**								
No	15/1479 (1.0)	Reference	Omitted		66/1158 (5.7)	Reference	Omitted	
Yes	1/113 (0.9)	0.9 (0.1–6.7)			8/53 (15.1)	**2.9 (1.3–6.5)**		

• Abbreviations: OR (Odds ratio), CI (Confidence interval), NS (Not significant).

• OR (95% CI) statistically significant at p< 0.1 on univariate and p<0.05 on multivariable logistic regression analysis presented in boldface.

• Tuberculosis infection, Malnutrition (reported, underweight), Pneumococcal infection on lytA PCR omitted from the multivariable logistic regression analysis due to lack of data.

• Use of supplementary oxygen therapy omitted due to collinearity with in-hospital death.

### Comparison of children who died at the rural hospitals vs. the urban hospital

In our comparison of urban and rural hospitals among children who died in-hospital, we found that children at the rural hospitals were more frequently of black race (99% vs. 88% in urban hospital, p = 0.002) and had antibiotics prescribed on admission (98% compared with 90% in the urban hospital, p = 0.025). We further found that children who died during hospitalization in the rural hospital (60%) were hospitalized for < 5 days compared to children from the urban hospital (41%), p = 0.029 (Table 5).

**Table 5:** Factors associated with hospital site (rural vs. urban) among children aged <5 years hospitalized with SARI who died in hospital, South Africa, 2009–2013.

Characteristics		Total	Urban hospital	Rural hospitals	P value
		N = 154 (%)	N = 51 (%)	N = 103 (%)	
**Socio-demographics**					
Age group (years)	< 1	124 (80.5)	42 (82.4)	82 (79.6)	0.686
	1–4	30 (19.5)	9 (17.6)	21 (20.4)	
Sex	Female	86 (55.8)	26 (51.0)	60 (58.3)	0.392
	Male	68 (44.2)	25 (49.0)	43 (41.7)	
Race	Black	147 (95.5)	45 (88.2)	102 (99.0)	**0.006** *
	Other	7 (4.5)	6 (11.8)	1 (1.0)	
2 or more doses of pneumococcal vaccine	Yes	49 (59.8)	18 (72.0)	31 (54.4)	0.134
	No	33 (40.2)	7 (28.0)	26 (45.6)	
Type of housing	Bricks	132 (85.7)	35 (68.6)	97 (94.2)	**<0.001** *
	Iron sheeting	14 (9.1)	14 (27.5)	0 (0.0)	
	Others	8 (5.2)	2 (3.9)	6 (5.8)	
Number of people sleeping in a room	>2	146 (96.7)	46 (92.0)	100 (99.0)	**0.041** *
	≤2	5 (3.3)	4 (8.0)	1 (1.0)	
**Clinical presentation and course**					
Symptoms prior to admission (days)	≥2	84 (55.3)	33 (66.0)	51 (50.0)	0.062
	<2	68 (44.7)	17 (34.0)	51 (50.0)	
Antibiotics prescribed on admission	Yes	146 (95.4)	45 (90.0)	101 (98.1)	**0.025** *
	No	7 (4.6)	5 (10.0)	2 (1.9)	
Supplementary oxygen therapy	Yes	126 (82.4)	43 (86.0)	83 (80.6)	0.410
	No	27 (17.6)	7 (14.0)	20 (19.4)	
Duration of hospitalization (days)	<5	82 (53.6)	21 (41.2)	61 (59.8)	**0.029**
	≥5	71 (46.4)	30 (58.8)	41 (40.2)	
**Co-infections and underlying medical conditions**					
HIV status	Positive	47 (49.0)	17 (56.7)	30 (45.5)	0.308
	Negative	49 (51.0)	13 (43.3)	36 (54.5)	
Tuberculosis infection	Yes	4 (17.4)	2 (13.3)	2 (25.0)	0.589*
	No	19 (82.6)	13 (86.7)	6 (75.0)	
Malnutrition (reported)	Yes	4 (2.6)	1 (2.0)	3 (2.9)	1.000*
	No	148 (97.4)	49 (98.0)	99 (97.1)	
Malnutrition (Underweight)	Yes	15 (46.9)	3 (60.0)	12 (44.4)	0.645*
	No	17 (53.1)	2 (40.0)	15 (55.6)	
*Any other underlying illness	Yes	14 (9.2)	6 (12.0)	8 (7.8)	0.405
	No	138 (90.8)	44 (88.0)	94 (92.2)	
**Respiratory pathogens**					
Any respiratory virus	Yes	88 (62.9)	33 (66.0)	55 (61.1)	0.566
	No	52 (37.1)	17 (34.0)	35 (38.9)	
Pneumococcal infection on lytA PCR	Yes	9 (10.0)	1 (6.2)	8 (10.8)	1.000*
	No	81 (90.0)	15 (93.8)	66 (89.2)	

• Column percentage were calculated as a percent of all those with available data for the variables (i.e. not including missing).

• Variables statistically significant at p< 0.05 presented in boldface, p value* (Fisher’s exact test).

• *Any other underlying illness (any of chronic lung disease, asthma, renal disease, heart disease, neurological disease, diabetes).

• Missing values for covariates were as follows: 2 or more doses of pneumococcal vaccine (n = 72), Number of people sleeping in a room (n = 3), Symptoms prior to admission (days) (n = 2), Antibiotics prescribed on admission (n = 1), Supplementary oxygen therapy (n = 1), Duration of hospitalization (days) (n = 1), HIV (n = 58), Tuberculosis infection (n = 131), Malnutrition (reported) (n = 2), Malnutrition (Underweight) (n = 122), Any other underlying illness (n = 2), Any respiratory virus (n = 14), Pneumococcal infection on lytA PCR (n = 64).

### Survival estimates among children who died in the rural and urban sites

**Fig 2:**
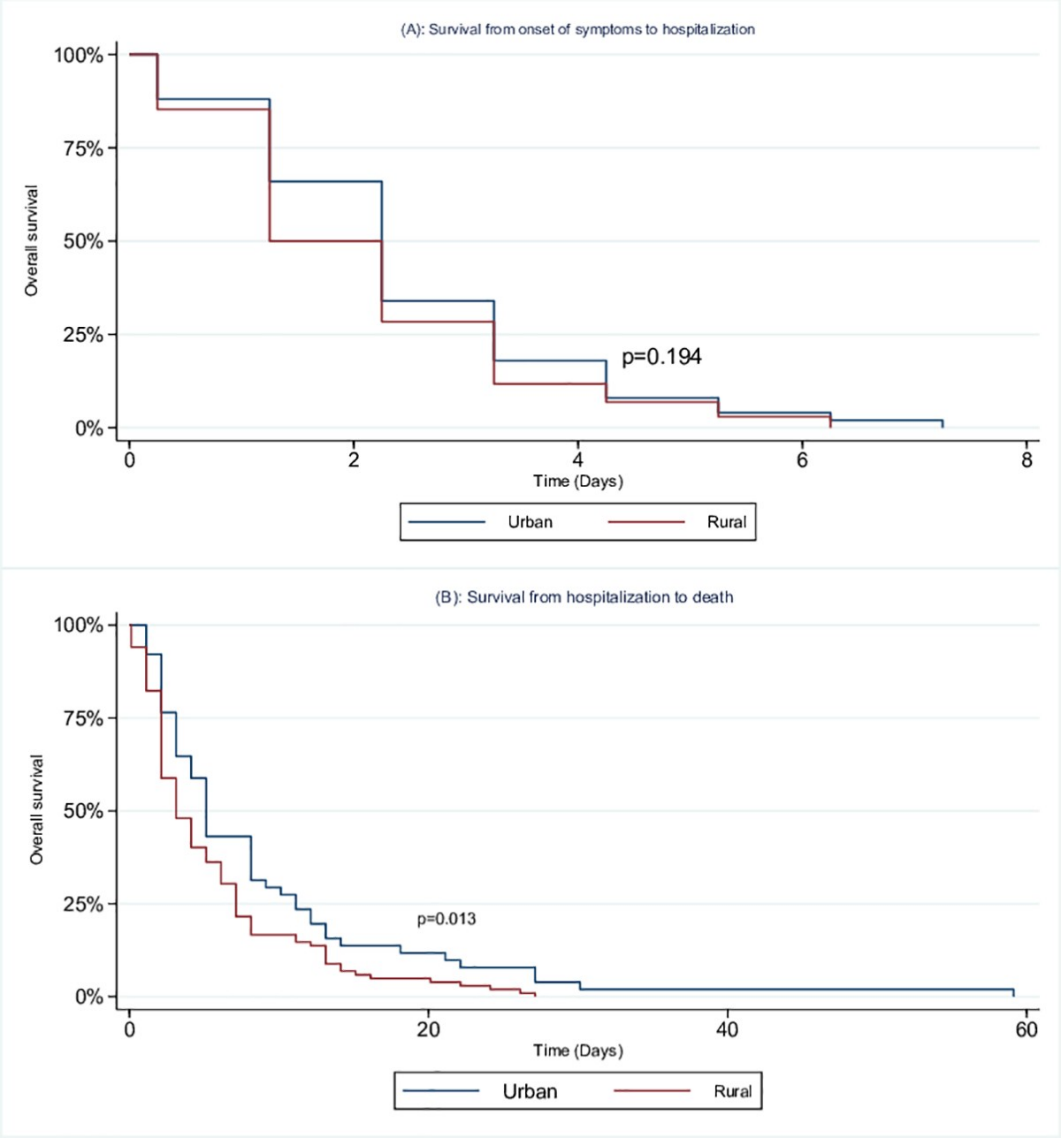
Kaplan-Meier survival estimates among children aged <5 years who died with SARI in urban and rural sites, South Africa, 2009 to 2013. (A) Survival from onset of symptoms to hospitalization (B) Survival from hospitalization to death.

Among children that died, the median duration of reported onset of symptoms to hospital admission was similar in the urban and rural hospitals (Fig 2A). The interval from date of hospitalization to death amongst those that died was shorter in the rural site (median 3 days (IQR 2–7)) compared to the urban site (median 5 days (IQR 3–11), p = 0.013) (Fig 2B).

## Discussion

We found that there was a substantially higher in-hospital mortality among children with SARI at the rural hospitals (6.9%) compared to the urban hospital (1.3%) in South Africa. HIV-infection was higher in the rural hospitals than the urban hospital, death was more common in HIV-infected children and correspondingly death was higher in the rural hospitals. HIV infection and presence of underlying illness were the factors associated with death. At the rural site, we found that HIV and younger age were independently associated with increased odds of death while individuals hospitalized for ≥ 5 days or with any respiratory virus detected were less likely to die. Survival function in terms of hospitalization to death amongst those who died was shorter at the rural site compared to the urban site.

While the 2.9% overall CFP reported in our study was higher than 1.7% reported in a case-series study among hospitalized children aged <5 years with SARI from 8 countries in Africa, the CFP estimate of our urban hospital is consistent [23]. Findings from the national demographic surveillance system in China and Bangladesh also recorded mortality that was 4.9 and 1.4 fold higher, **respectively**, in rural area compared to urban areas [24,25].

The difference in risk factors between urban and rural hospitals could be due to lower power to detect a significant difference for some factors in the urban area because of fewer deaths recorded. Higher mortality among children in rural hospitals could be due to several factors, including differences in standards of living, availability of or access to public health facilities and services [26]. Children in rural areas possibly present with more severe illness due to delayed hospitalization related to remoteness of rural villages and quality of in-hospital care [11,27]. In addition, it is possible that patients have earlier access to high-quality care even before admission, in the urban site, so reducing the impact of these factors on outcome [28]. However, delay in presentation to the hospital as a contributor to higher mortality in the rural site was not supported by the data because patients from the rural site reported a similar interval between symptom onset and hospitalization, but a shorter interval from hospitalization to death. Factors such as underlying medical conditions and quality of care delivered at each site could affect the CFP. However, there was no significant difference in the prevalence of other underlying conditions apart from HIV between the urban and rural hospitals, but in the urban hospital, other underlying illness was found to be associated with in-hospital death which is in keeping with studies that have shown that children with underlying chronic diseases are more likely to die in-hospital [29,30]. We were not able to assess quality of care in this study.

At both urban and rural hospitals, HIV was a risk factor for death. HIV-infected individuals had 11 times greater odds of in-hospital mortality than HIV-uninfected children in the urban hospital while in the rural hospitals HIV-infected individuals had 3 times greater odds of in-hospital mortality than HIV-uninfected children. This finding from our urban hospital was consistent with other studies carried out in South Africa, which estimated approximately 10 times elevated mortality risk in HIV-infected children and with global estimates [5,31]. Amongst those that died at the urban site, 57.7% were HIV-infected while at the rural area 45.5% were HIV-infected. There have been studies showing that reduction in SARI is associated with availability of ARV but despite these studies a high incidence of SARI is still reported in HIV-infected children on ARV [32–34].

While the HIV prevalence declined at both urban and rural hospitals over the study period, the prevalence at the rural hospitals was consistently twice that of the urban hospital (Table 3), reflecting the underlying community prevalence. Goga et al conducted an evaluation of community-specific HIV prevalence which showed a higher prevalence among infants and pregnant women, and higher rates of mother to child transmission in Mpumalanga Province (rural hospitals) (5.7%, 95% CI 4.1–7.3) compared to Gauteng Province (urban hospital) (2.5%, 95% CI 1.5–3.6) [35].

We demonstrated that in our rural hospitals children aged <1 year had a higher CFP compared to children aged 1–4 years. Several studies have found higher all-cause mortality in children aged <1 year mainly due to their immature immune system and vulnerability to infections [12,36–39]. We found that in our rural hospitals, children hospitalized for 5 or more days were less likely to die compared to those hospitalized for <5 days. A study carried out in a developing country also found that over 50% of death among children with pneumonia occurred within the first four days of hospitalization [40]. This finding is a contrasting finding compared to several other studies which show that there is higher probability of dying from a wide range of diseases including influenza, covid-19 and heart failure with a longer hospital stay [41–43]. Detection of a respiratory virus was significantly associated with lower mortality. A multi-country African similarly study found lower CFP in patients testing influenza positive [23], and further studies have associated bacterial aetiologies more commonly with death as these are often severe [44–46]. Similar to these studies, among children tested with pneumococcal lytA PCR, CFP was higher among those with versus those without pneumococcal infection (5% vs. 3%); however, this difference was not statistically significant.

Detection of pneumococcus by *lytA* PCR of a blood sample was low at both sites (7.1% in our rural site and 4.4% in our urban site), likely related to lower sensitivity of available diagnostic assays for pneumococcal pneumonia, infection with pneumococcus was not evaluated in the multivariable model due to high number of missing data. However, studies from South Africa, India, global estimates, and systemic reviews have reported positive associations of pneumococcal infection with death in children with SARI [6,44,45,47]. In our study, vaccination with 2 or more doses of PCV was significantly higher in the urban site compared to the rural site. Despite advances in preventing SARI caused by *S*. *pneumoniae* with the introduction of PCV into the Expanded Program on Immunization (EPI) in 2009, pneumococcus remains a relatively common cause of death in children with SARI in South Africa.

Our study has limitations. First, cases from only three hospitals were used for this study; therefore, our findings might not be generalizable to other rural and urban hospitals. Second, our study enrolment could not account for children who died at home, or died too soon after presentation to be enrolled, which might lead to underestimation of mortality. Third, though our enrolment forms account for many common factors associated with in-hospital death, we were unable to collect data on some possible confounders, such as indoor air pollution, socio-economic status and type of feeding. Fourth, we were not able to assess the quality of care at each hospital including one rural hospital which is a district hospital. Lastly, malnutrition, CD4+ T-lymphocyte count, and ART use were inconsistently collected and could not be included in our model.

In conclusion, our study suggests a higher mortality in children hospitalized with SARI at two rural hospitals compared to an urban hospital. Without question, HIV remains a major risk factor for SARI mortality, and wider availability of MTCT services is needed to help reduce the disease burden. But further studies are urgently needed to identify the causal factors leading to an inherently higher risk of mortality in our rural hospitals, such as differences in treatment or access to care and health care inequality.

## Supporting information

S1 Table Comparison of the demographic characteristics, clinical presentation and respiratory pathogens detected among children aged <5 years hospitalized with SARI at rural hospitals (Mapulaneng and Matikwana), South Africa 2009–2013. (DOCX)

S2 Table Comparison of the demographic characteristics, clinical presentation and respiratory pathogens detected among children aged <5 years with SARI in urban and rural hospitals, South Africa 2009–2013. Sensitivity analysis including children admitted in the stay over ward. (DOCX)

S3 Table Demographics and clinical characteristics of HIV-infected and HIV-uninfected children aged <5 years hospitalized with SARI by hospital site, South Africa 2009–2013. (DOCX)

S4 Table Percentage of children HIV-infected by year compared with sensitivity analysis (assuming all those with missing data were HIV-uninfected) amongst children aged <5 years hospitalized with SARI in urban and rural hospital sites, South Africa, 2009–2013. (DOCX)
