# Supplementary material for: Mortality in children aged <5 years with severe acute respiratory illness in a high HIV-prevalence urban and rural areas of South Africa, 2009–2013
Source: PLoS One. 2021 Aug 12;16(8):e0255941. doi: 10.1371/journal.pone.0255941 (PMC8360538; doi:10.1371/journal.pone.0255941)
Supplement: S1 Table — (DOCX) [file pone.0255941.s001.docx]

**S1 table: Comparison of the demographic characteristics, clinical presentation and respiratory pathogens detected among children aged <5 years hospitalized with SARI at rural hospitals (Mapulaneng and Matikwana), South Africa 2009–2013.**

| **Characteristics** | **Total** | **Mapulaneng** | **Matikwana** | **P value** |
| --- | --- | --- | --- | --- |
|  | **N=1486** | **N=608** | **N= 878** |  |
| **Socio-demographics** | **n/N (%)** | **n/N (%)** | **n/N (%)** |  |
| Age group <1 year | 874/1486 (58.8) | 292/608 (48.0) | 582/878 (66.3) | **<0.001** |
| Sex (Female) | 641/1486 (43.1) | 270/608 (44.4) | 371/878 (42.3) | 0.410 |
| Black race | 1479/1486 (99.5) | 606/608 (99.7) | 873/878 (99.4) | 0.506 |
| 2 or more doses of pneumococcal vaccine | 551/855 (64.4) | 183/324 (56.5) | 368/531 (69.3) | **<0.001** |
| Type of housing |  |  |  |  |
| Bricks | 1419/1486 (95.5) | 565/608 (92.9) | 854/878 (97.3) | **<0.001** |
| Iron sheeting | 7/1486 (0.5) | 5/608 (0.8) | 2/878 (0.2) |  |
| Others | 60/1486 (4.0) | 38/608 (6.3) | 22/878 (2.5) |  |
| > 2 people sleeping in a room | 1450/1465 (99.0) | 587/593 (99.0) | 863/872 (99.0) | 0.970 |
| **Clinical presentation and course** |  |  |  |  |
| Symptoms ≥2 days prior to admission | 668/1474 (45.3) | 336/600 (56.0) | 332/874 (38.0) | **<0.001** |
| Antibiotics prescribed on admission | 1469/1479 (99.3) | 599/604 (99.2) | 870/875 (99.4) | 0.554 |
| Supplementary oxygen therapy | 312/1474 (21.2) | 109/600 (18.2) | 203/874 (23.2) | **0.019** |
| Duration of hospitalization (days) |  |  |  |  |
| <5 | 722/1466 (49.2) | 313/598 (52.3) | 409/868 (47.1) | **0.049** |
| ≥5 | 744/1466 (50.8) | 285/598 (47.7) | 459/868 (52.9) |  |
| In-hospital death | 103/1486 (6.9) | 36/608 (5.9) | 67/878 (7.6) | 0.202 |
| **Co-infections and underlying medical conditions** |  |  |  |  |
| HIV-infection | 233/1126 (20.7) | 97/441 (22.0) | 136/685 (19.9) | 0.387 |
| Tuberculosis infection | 7/123 (5.7) | 2/33 (6.1) | 5/90 (5.6) | 0.915 |
| Malnutrition (reported) | 7/1482 (0.5) | 0/604 (0.0) | 7/878 (0.8) | **0.028** |
| Malnutrition (Underweight) | 99/459 (22.0) | 30/160 (18.8) | 69/299 (23.1) | **0.283** |
| *Any other underlying illness | 61/1482 (4.1) | 24/604 (4.0) | 37/878 (4.2) | 0.819 |
| **Respiratory pathogens** |  |  |  |  |
| Any respiratory virus | 1082/1426 (75.9) | 476/600 (79.3) | 606/826 (73.4) | **0.009** |
| Pneumococcal infection on lytA PCR | 53/1211 (4.4) | 18/483 (3.7) | 35/728 (4.8) | 0.368 |

- *Column percentage were calculated as a percent of all those with available data for the variables (i.e. not including missing)*
- *Variables statistically significant at p< 0.05 presented in boldface.*
- **Any other underlying illness (any of chronic lung disease, asthma, renal disease, heart disease, neurological disease, diabetes)*
