# Supplementary material for: Mortality in children aged <5 years with severe acute respiratory illness in a high HIV-prevalence urban and rural areas of South Africa, 2009–2013
Source: PLoS One. 2021 Aug 12;16(8):e0255941. doi: 10.1371/journal.pone.0255941 (PMC8360538; doi:10.1371/journal.pone.0255941)
Supplement: S2 Table — (DOCX) [file pone.0255941.s002.docx]

**S2 table: Comparison of the demographic characteristics, clinical presentation and respiratory pathogens detected among children aged <5 years with SARI in urban and rural hospitals, South Africa 2009–2013. Sensitivity analysis including children admitted in the stay over ward.**

| **Characteristics** | **Total** | **Urban hospital** | **Rural hospitals** | **P value** |
| --- | --- | --- | --- | --- |
|  | **N=8069** | **N=6583** | **N= 1486** |  |
|  | n/N (%) | n/N (%) | n/N (%) |  |
| Age <1 year | 5226/8069 (64.8) | 4352/6583 (66.1) | 874/1486 (58.8) | **<0.001** |
| Sex (Female) | 3396/8069 (42.1) | 2755/6583 (41.9) | 641/1486 (43.1) | 0.365 |
| Black race | 7911/8069 (98.0) | 6432/6583 (97.7) | 1479/1486 (99.5) | **<0.001** |
| Type of housing |  |  |  | **<0.001** |
| Brick | 6171/8069 (76.5) | 4752/6583 (72.2) | 1419/1486 (95.5) |  |
| Iron sheeting | 1765/8069 (21.9) | 1758/6583 (26.7) | 7/1486 (0.5) |  |
| Other | 133/8069 (1.6) | 73/6583 (1.1) | 60/1486 (4.0) |  |
| 2 or more doses of pneumococcal vaccine | 2417/3288 (73.5) | 1866/2433 (76.7) | 551/855 (64.4) | **<0.001** |
| >2 people sleeping in a room | 7501/7985 (93.9) | 6051/6520 (92.8%) | 1450/1465 (99.0) | **<0.001** |
| **Clinical presentation** |  |  |  |  |
| Symptoms ≥2 days prior to admission | 4344/8020 (54.2) | 3676/6546 (56.2) | 668/1474 (45.3) | **<0.001** |
| Antibiotics prescribed on admission | 7191/7846 (91.7) | 5722/6367 (89.9) | 1469/1479 (99.3) | **<0.001** |
| Supplemental oxygen therapy | 2422/7988 (30.3) | 2110/6514 (32.4) | 312/1474 (21.2) | **<0.001** |
| Duration of hospitalization (days) |  |  |  |  |
| <5 | 5269/7981 (66.0) | 4547/6515 (69.8) | 722/1466 (49.2) | **<0.001** |
| ≥5 | 2712/7981 (34.0) | 1968/6515 (30.2) | 744/1466 (50.8) |  |
| In-hospital death | 157/8069 (1.9) | 54/6583 (0.8) | 103/1486 (6.9) | **<0.001** |
| **Underlying medical conditions** |  |  |  |  |
| HIV infection | 568/5370 (10.6) | 335/4244 (7.9) | 233/1126 (20.7) | **<0.001** |
| Tuberculosis | 103/1116 (9.2) | 96/993 (9.7) | 7/123 (5.7) | 0.151 |
| Malnutrition (reported) | 34/8048 (0.4) | 27/6566 (0.4) | 7/1482 (0.5) | 0.743 |
| Malnutrition (underweight) | 356/1614 (22.1) | 257/1155 (22.3) | 99/459 (21.6) | 0.765 |
| *****Any other underlying illness | 344/8049 (4.3) | 283/6567 (4.3) | 61/1482 (4.1) | 0.740 |
| **Respiratory pathogens** |  |  |  |  |
| Any respiratory virus | 6427/7878 (81.6) | 5345/6452 (82.8) | 1082/1426 (75.9) | **<0.001** |
| Pneumococcal infection on *lytA* PCR | 237/4455 (5.3) | 184/3244 (5.7) | 53/1211 (4.4) | 0.087 |

*Column percentage were calculated as a percent of all those with available data for the variable (i.e. not including missing)*

**Any other underlying illness (any of chronic lung disease, asthma, renal disease, heart disease, neurological disease, diabetes)*
