# Supplementary material for: Mortality in children aged <5 years with severe acute respiratory illness in a high HIV-prevalence urban and rural areas of South Africa, 2009–2013
Source: PLoS One. 2021 Aug 12;16(8):e0255941. doi: 10.1371/journal.pone.0255941 (PMC8360538; doi:10.1371/journal.pone.0255941)
Supplement: S3 Table — (DOCX) [file pone.0255941.s003.docx]

**S3 table: Demographics and clinical characteristics of HIV-infected and HIV-uninfected children aged <5 years hospitalized with SARI by hospital site, South Africa 2009–2013**

|  | **Urban hospital** | | | **Rural hospitals** | | |
| --- | --- | --- | --- | --- | --- | --- |
| **Characteristics** | **HIV-infected** | **HIV-uninfected** | **P value** | **HIV-infected** | **HIV-uninfected** | **P value** |
|  | **N=257** | **N=2236** |  | **N=233** | **N=893** |  |
|  | **n/N (%)** | **n/N (%)** |  | **n/N (%)** | **n/N (%)** |  |
| Age group <1 year | 189/257 (73.5) | 1691/2236 (75.6) | 0.462 | 124/233 (53.2) | 532/893 (59.6) | 0.080 |
| Sex (Female) | 123/257 (47.9) | 939/2236 (42.0) | 0.072 | 119/233 (51.1) | 351/893 (39.3) | **0.001** |
| Black race | 250/257 (97.3) | 2191/2236 (98.0) | 0.450 | 232/233 (99.6) | 889/893 (99.6) | 0.969 |
| 2 or more doses of pneumococcal vaccine | 69/97 (71.1) | 637/846 (75.3) | 0.371 | 74/125 (59.2) | 374/561 (66.7) | 0.114 |
| >2 people sleeping in a room | 236/256 (92.2) | 2046/2205 (92.8) | 0.726 | 228/230(99.1) | 871/881 (98.9) | 0.729 |
| **Clinical presentation and course** |  |  |  |  |  |  |
| Symptoms ≥2 days prior to admission | 162/254 (63.8) | 1308/2221 (58.9) | 0.133 | 132/232 (56.9) | 367/889 (41.3) | **<0.001** |
| Antibiotics prescribed on admission | 240/251 (95.6) | 2026/2165 (93.6) | 0.205 | 231/231(100.0) | 882/890 (99.1) | 0.148 |
| Supplementary oxygen therapy | 171/254 (67.3) | 1181/2216 (53.3) | **<0.001** | 58/230 (25.2) | 156/890 (17.5) | **0.008** |
| Duration of hospitalization (days) |  |  |  |  |  |  |
| <5 days | 71/254 (28.0) | 1081/2214 (48.8) | **<0.001** | 71/229 (31.0) | 467/886 (52.7) | **<0.001** |
| ≥5 days | 183/254 (72.0) | 1133/2214 (51.2) |  | 158/229 (69.0) | 419/886 (47.3) |  |
| In-hospital death | 17/257 (6.6) | 13/2236 (0.6) | **<0.001** | 30/233 (12.9) | 36/893 (4.0) | **<0.001** |
| **Co-infections and underlying medical conditions** |  |  |  |  |  |  |
| Tuberculosis infection | 19/79 (24.1) | 47/514 (9.1) | **<0.001** | 2/37 (5.4) | 3/65 (4.6) | 0.859 |
| Malnutrition (reported) | 6/256 (2.3) | 10/2234 (0.4) | **<0.001** | 1/233 (0.4) | 3/892 (0.3) | 0.832 |
| Malnutrition (underweight) | 15/33 (45.5) | 121/470 (25.7) | **0.014** | 22/49 (44.9) | 53/298 (17.8) | **<0.001** |
| *Any other underlying illness | 14/256 (6.6) | 115/2235 (5.1) | 0.825 | 13/233 (5.6) | 33/892 (3.7) | 0.197 |
| **Respiratory pathogens** |  |  |  |  |  |  |
| Any respiratory virus | 183/257 (71.2) | 1769/2222 (79.6) | **0.002** | 145/228 (63.6) | 695/876 (79.3) | **<0.001** |
| Pneumococcal infection on *lytA* PCR | 16/138 (11.6) | 80/1210 (6.6) | **0.031** | 19/219 (8.7) | 24/829 (2.9) | **<0.001** |

- *Column percentage were calculated as a percent of all those with available data for the variables (i.e. not including missing), Variables statistically significant at p< 0.05 presented in boldface, *Any other underlying illness (any of chronic lung disease, asthma, renal disease, heart disease, neurological disease, diabetes)*
