# Supplementary material for: Mortality in children aged <5 years with severe acute respiratory illness in a high HIV-prevalence urban and rural areas of South Africa, 2009–2013
Source: PLoS One. 2021 Aug 12;16(8):e0255941. doi: 10.1371/journal.pone.0255941 (PMC8360538; doi:10.1371/journal.pone.0255941)
Supplement: S4 Table — (DOCX) [file pone.0255941.s004.docx]

**S4 table: Percentage of children HIV-infected by year compared with sensitivity analysis (assuming all those with missing data were HIV-uninfected) amongst children aged <5 years hospitalized with SARI in urban and rural hospital sites, South Africa, 2009–2013.**

|  | **HIV-infected** | | | **HIV-infected (sensitivity analysis)** | | |
| --- | --- | --- | --- | --- | --- | --- |
| **Year** | **Urban site N=2493**  **n/N (%)** | **Rural site N=1126**  **n/N (%)** | **P value** | **Urban site N=3811**  **n/N (%)** | **Rural site N=1486**  **n/N (%)** | **P value** |
| 2009 | 102/655 (16) | 53/166 (32) | <0.001 | 102/1031 (10) | 53/330 (16) | 0.002 |
| 2010 | 62/503 (12) | 73/262 (28) | <0.001 | 62/791 (8) | 73/303 (24) | <0.001 |
| 2011 | 37/513 (7) | 35/220 (16) | <0.001 | 37/795 (5) | 35/252 (14) | <0.001 |
| 2012 | 44/559 (8) | 46/259 (18) | <0.001 | 44/793 (6) | 46/306 (15) | <0.001 |
| 2013 | 12/263 (5) | 26/219 (12) | 0.003 | 12/401 (3) | 26/295 (9) | 0.001 |
| Overall | 257/2493 (10) | 233/1126 (21) | <0.001 | 257/3811 (7) | 233/1486 (16) | <0.001 |
